# Effects of a Meal on the Hemorheologic Responses to Exercise in Young Males

**DOI:** 10.1155/2014/862968

**Published:** 2014-06-26

**Authors:** Jan Bilski, Aneta Teległów, Janusz Pokorski, Jacek Nitecki, Joanna Pokorska, Ewa Nitecka, Anna Marchewka, Zbigniew Dąbrowski, Jakub Marchewka

**Affiliations:** ^1^Department of Ergonomics and Exercise Physiology, Faculty of Health Sciences, Jagiellonian University Medical College, 31-531 Cracow, Poland; ^2^Department of Clinical Rehabilitation, University School of Physical Education, 31-571 Cracow, Poland; ^3^Joseph Dietl Hospital, 31-121 Cracow, Poland

## Abstract

*Aim*. This study investigates the changes in hemorheologic parameters resulting from exercise followed by a standard meal. *Methods*. In twelve moderately active men a period of exercise on a bicycle ergometer for 30 min at 60% VO_2max_ was followed by a test meal or by 30 min rest. Venous blood was sampled for further analysis at baseline, after exercise, and after the meal/rest period. *Results*. The elongation index (EI) was reduced and a marked rise in plasma viscosity was observed after exercise. A significant decrease in half time of total aggregation (*T*_1/2_) and a rise in aggregation index (AI) after exercise were observed; however, after the postexercise period these changes were reversed. *Conclusion*. The present study demonstrates that physical exercise causes several changes in blood rheology parameters, such as an increase of blood viscosity, a decrease in EI and an increase in AI, and a fall in the *T*_1/2_ values. The meal eaten in the postexercise period caused a further reduction in EI values indicating higher red cell rigidity, but not in plasma viscosity or aggregations indices. Such alterations in hemorheologic parameters should not impair the function of the cardiovascular system in fit and healthy people but it could constitute a serious risk under various pathophysiological conditions.

## 1. Introduction

The importance of physical activity in preventing most chronic cardiovascular and metabolic diseases and the role of exercise as an adjunctive therapy regime is widely accepted. Generally, the beneficial effects of exercise are associated with metabolic improvement and neutralization of some of the risk factors connected with a sedentary lifestyle [1]. The protective effect of exercise may also be attributed to its anti-inflammatory effect which is at least partly caused by muscle-derived peptides, the so-called “myokines” [2]. The contracting skeletal muscles release myokines with endocrine effects mediating direct anti-inflammatory effects and/or specific effects on visceral fat [2]. Systematic exercise could lead to some adjustments of the rheological properties of blood that could potentially be involved in its beneficial effects [3, 4].

However, it should be considered that intensive, acute exercise may sometimes constitute a risk of acute cardiovascular accidents. Acute exercise leads to several changes in blood rheological parameters, including increased blood viscosity. A sudden rise in blood viscosity may impair the microcirculatory blood flow and oxygen supply to the tissues. Increased blood viscosity is treated as an important risk factor for cardiovascular and cerebrovascular disorders; decreased deformability and increased aggregation of red blood cells (RBC) were reported in such diseases as hypertension, diabetes mellitus (DM), and coronary artery disease [3, 5–9].

Rheological properties of blood are influenced by several factors, including metabolic and humoral factors [10]. It is possible that nutritional factors could also influence the rheological changes associated with exercise but these effects are relatively understudied and only observed in a specific model when a meal preceded exercise [11, 12]. It was suggested, however, that the meal which was consumed (particularly the carbohydrate component) not only influences the rheological blood properties but also accelerates myocardial ischemia, reduces exercise capacity, and is associated with a more rapid increase in the determinants of myocardial oxygen consumption [12]. An increased food intake in response to acute exercise was reported and is considered to be the result of cognitive factors including attitudes and beliefs associated with exercise, such as the common behavior of using food as a reward for exercising [13]. To our knowledge the effects of a meal following exercise on hemorheologic parameters, such as viscosity, deformability, and increased aggregation of red blood cells, were not tested.

The aim of this study was to analyze the changes in hemorheologic parameters resulting from exercise followed by a standard meal.

## 2. Material and Methods

### 2.1. Participants

Twelve moderately active, nonsmoking male volunteers (mean ± SEM, age = 28.7 ± 3.7; height = 1.86 ± 0.1 m; weight = 78.2 ± 9.7, body mass index (BMI) 22.6 ± 0.3 kg/m^2^) were recruited to participate in this study. None of the subjects had signs or symptoms of acute or chronic disease or took any medication. Volunteers received monetary compensation for their participation. They were fully informed of the study details and expressed their written consent prior to taking part in the study. The study protocol was approved by the Jagiellonian University Bioethics Committee, and all the procedures complied with the Declaration of Helsinki.

### 2.2. Study Protocol

All subjects were instructed to consume a staple diet containing 40–50% carbohydrates, 15–25% proteins, and 30–40% fat (energy percent) at least two weeks prior to and throughout the study period. They were asked to refrain from taking vigorous exercise and ingesting caffeine or alcohol 24 h prior to the main trials. All experiments started at 8.00 a.m., after a 12-hour overnight fast.

Subjects attended the laboratory for an initial session during which anthropometric data was collected and they were familiarized with the equipment. A submaximal fitness test was used to estimate VO_2max⁡_, bearing in mind the considerable limitations of that approach [14]. The test was performed on a Monark cycle ergometer with the seat height adjusted so that the subject's knee was slightly flexed when the ball of the foot rested on the pedal at the lowest point in a revolution. Heart rates were monitored and recorded using a HR monitor (Polar F1—Polar Electro Oy, Kempele, Finland). The ambient temperature range was about 23.8°C. Initial work loads of 120 W at 60 pedal revs. per min were used. If the heart rate after 2 minutes was less than 120 bpm, the work load was increased to 180 W and the test was continued. If in the 4th minute the heart rate was less than 120 bpm, the work load was increased to 210 W and the test was continued until that criterion was met. The predicted VO_2max⁡_ was read from a nomogram or accompanying tables and multiplied by the von Dobeln age correction factors [15, 16].

During actual intervention, the subjects underwent two test sessions: a high intensity exercise on a bicycle ergometer for a 30 min period at 60% predicted VO_2max⁡_ followed by a test meal (test meal series) or by a period of 30 min of rest (control series) in random order. During the test meal the subjects received sandwiches consisting of bread, butter, and ham (2.73 kcal/g, energy percent: 44.4% carbohydrates, 16.2% proteins, and 39.4% fat) which they were supposed to consume until reaching satiety. The two test sessions were performed on two separate days, with a minimal period of 14 days between the sessions. The participants had free access to water in the postexercise period.

Blood samples were collected into EDTA tubes from the antecubital vein at baseline, after exercise, and after eating. The following basic hematological parameters were determined: (1) red blood cell count (RBC, 10^6^/mm^3^), (2) hemoglobin (Hb, g/dL), and (3) hematocrit (HCT, %).

### 2.3. Rheological Analysis

The rheological analysis was conducted as described above [17, 18]. The deformability and aggregation of red blood cells were determined using a laser-assisted optical rotational cell analyzer (LORCA, RR Mechatronics, Holland) according to Hardeman's method [7, 8]. The deformability was expressed using the elongation index (EI). Additionally, the maximum EI (EI_max⁡_) and the shear stress required to cause one half of this maximum (SS_1/2_) were determined using the Lineweaver-Burk approach and nonlinear curve fitting using a nonlinear curve-fitting algorithm available in a commercial statistical package (Prism 6.2, GraphPad Software Inc., La Jolla, CA). The SS_1/2_/EI_max⁡_ ratio was calculated as a normalized measure of SS_1/2_. SS_1/2_/  EI_max⁡_ is inversely related to RBC deformability such that a lower value indicates better deformability. Details and justification of this method have been described elsewhere [18, 19].

The following aggregation parameters were estimated: (1) aggregation index (AI, %), (2) the amplitude and total extent of aggregation (AMP, arbitrary units), and (3) the half time (*T*_1/2_, s) which describes the kinetics of the aggregation process and which is proportional to the time of reaggregation of the disintegrated red cell complexes. Measurements of aggregation parameters were carried out at native hematocrit. The temperature in the LORCA was adjusted to 37°C. All other preparations and measurements were carried out at room temperature (22 ± 1°C).

For each measurement of deformability, a 25 *μ*L blood sample was prepared in 5 mL of a 0.14 mM polyvinylpyrrolidone (PVP, *M* = 360,000, *η* = 31.8 mPa·s at 37°C in PBS) solution. The samples were injected into the LORCA measuring system, where they were subjected to varying levels of shear stress. That process was fully automated (a shear stress between 0.30 Pa and 59.97 Pa was applied). The EI for erythrocytes was calculated according to the formula:

(1) $$\begin{array}{c} \text{EI}=\frac{\left(A-B\right)}{\left(A+B\right)}, \end{array}$$

where *A* and *B* represent the vertical and horizontal axes of the ellipsoid, respectively. EI allowed for the estimation of erythrocyte elasticity and was calculated based on the change in erythrocyte shape (from round to ellipsoid) under the influence of shear stress.

Aggregation measurements obtained from the LORCA aggregometer were based on the detection of laser backscattering from the sheared (disaggregated) and unsheared (aggregating) blood using a computer-assisted system. Each 2 mL sample of blood was transferred into a glass vessel and oxygenated for 10 to 15 min prior to obtaining measurements. A 1 mL sample of blood was injected into the gap between the outer cylinder “cup” and inner cylinder “bob” of the LORCA. During the measurement, the cup was driven by a computer-controlled stepper motor. The blood sample was sheared at 400 s^−1^, with the shear rate decreasing rapidly to zero. The backscattering data was evaluated by the computer and the AI was calculated from the syllectrogram (light scatter versus time curve during a 120 s period). This method relies on the fact that there is less light backscattered from aggregating red cells.

### 2.4. Other Measurements

The viscosity of plasma was determined in a viscosimeter (type D-52159 Roetgen, Myrenne, Germany) with results displayed in mPa · s. Concentrations of fibrinogen were determined with a Chrom 7 coagulometer (Slamed Ing GmbH, Germany), with results displayed as g/L plasma lactate concentration ([La]pl) and measured using an automatic analyzer (Ektachem XR 700, Kodak, USA).

### 2.5. Statistical Analysis

Results are presented as mean and SD or as median and interquartile range according to the distribution of the data. The Kolmogorov-Smirnov test was used to evaluate the normal distribution of the continuous data. The paired Student's* t*-test or the Wilcoxon matched-paired ranked-signs test was used to compare the changes between measurements. Student's* t*-test was used to compare means between the meal and the control series of normally distributed variables. For comparison of the not normally distributed variables the nonparametric Mann-Whitney *U* test was used. *P* < 0.05 was considered statistically significant. All of the above statistical analyses were performed using SPSS version 20 for Windows.

## 3. Results

### 3.1. Hematological Parameters

There was no significant change detected in the values of RBC and Hb between measurements and these results were omitted for the sake of clarity. HCT values increased significantly after exercise but returned to normal within the next 30 min in both series as shown on Tables 1 and 2.

When comparing HCT values between the experimental series we observed that after the test meal they were significantly higher than after the rest period in the control series (*P* = 0.001).

### 3.2. Deformability Measurements

RBC deformability (assessed as the elongation index, EI) was found to be significantly reduced immediately after exercise at shear stress levels of 0.58 Pa–31.04 Pa (Tables 3 and 4).

This effect was sustained after exercise during the 30 min rest period in the control series, but after the test meal we observed a further statistically significant reduction in median EI at shear stress levels 8.23 (Pa)–59.97 (Pa) (Tables 3 and 4).

Effects of exercise on EI_max⁡_, SS_1/2,_ and SS_1/2_/EI_max⁡_ are presented in Tables 5 and 6. We observed a significant increase in SS_1/2 _value and decrease in SS_1/2_/EI_max⁡_ ratio after exercise but not after meal or rest period.

### 3.3. Aggregation Indices and Plasma Viscosity

We observed similar, marked, and significant rises in plasma viscosity immediately after exercise in both series (Tables 1 and 2) and in the control series we observed further significant increases in PV (Table 2) during the 30 min rest period. This effect, however, was not observed after the test meal as shown in Table 1. We also observed significant decreases in half time of total aggregation (*T*_1/2_) and significant rises in aggregation index (AI) immediately after exercise similarly in both series as shown on Tables 3 and 4, but after the 30-minute rest period in the control series, these changes were reversed (Table 4). Similarly, a statistically significant but less pronounced effect was seen after the test meal as shown in Table 3.

### 3.4. Other Measurements

The exercise caused a pronounced and significant increase in plasma lactate in both series as shown on Tables 1 and 2. Within the next 30 min we observed a slight decrease, similar to the postprandial and rest periods, but this effect was not statistically significant (Tables 1 and 2). There was a slight but statistically significant increase in fibrinogen plasma levels after exercise in the test meal series (Table 1). On the other hand we observed a significant decrease in fibrinogen plasma levels after the next 30 min both in the test meal and control series (Tables 1 and 2).

## 4. Discussion

Until now, no study had investigated the effects of a meal consumed after intensive exercise on hemorheologic factors (i.e., blood viscosity, RBC deformability, and aggregation properties).

We have demonstrated a sharp increase of plasma viscosity following high intensity exercise accompanied by a hematocrit elevation. A postexercise rise in blood viscosity is a well-reported phenomenon. Hematocrit and plasma viscosity are two of the major determinants of blood viscosity. There are several mechanisms responsible for a postexercise rise in blood viscosity: a fluid shift, a rise in the number of circulating erythrocytes due to splenic contraction and their redistribution, an increase of plasma protein concentration, and loss of water caused by thermoregulatory mechanisms and its entrapment in muscle cells [3]. Considering oxygen supply to the tissue, changes in plasma viscosity are more important because the increase in hematocrit, although reducing blood flow, increases oxygen carrying capacity [20]. Rise in plasma viscosity is associated with a rise in plasma proteins and particularly fibrinogen [4].

Changes to rheological properties of blood could depend on the type of exercise. Different effects of cycling and running on blood rheology were observed. Cycling exercise similar to that used in our study leads to a rise in blood viscosity, due to alterations in plasma viscosity and hematocrit. Outdoor running, however, does not increase blood viscosity or hematocrit [21–23].

It is of interest that we observed further increases in plasma viscosity after the 30 min rest period in the control series, but not after the test meal. Although participants in both groups had free access to water in the postexercise period, maybe those consuming a meal were better hydrated. It has been recommended that the sampling should be done after overnight fasting for the determination of blood and plasma viscosity [24] but several postprandial studies reported a decrease in viscosity suggesting relative overnight dehydration [25, 26]. However in active healthy people moderate dehydration occurring during exercise has only a slight effect on blood viscosity [27, 28].

We also observed significant changes in erythrocytes deformability after exercise, as shown by a statistically significant decrease in EI and rise in SS_1/2_/EI_max⁡_ ratio. This is in agreement with several studies demonstrating that physical exercise could also affect rheological properties of erythrocytes. However, it was shown that these alterations were not observed when red cell rheology was investigated after resuspension of cells on a buffer, which indicates that those changes were mostly due to plasma factors rather than to intrinsic red cell properties [3, 29].

In the present study we also demonstrated a marked increase in plasma lactate levels after exercise. Increased lactate could be, at least in part, responsible for observed changes in deformability. It was shown that in experimental conditions lactate shrinks the red cells and decreases their flexibility. The correlation between lactate concentrations and red cell rigidity after exercise intervention has been shown in some studies [3, 29]. It is, however, of interest that in highly trained athletes after intensive exercise an increase in red cell flexibility in spite of high blood lactate levels does not rigidify the red cell, unlike in sedentary or in the moderately active subjects [30, 31]. It is noteworthy that when exercise in the present study was followed by a meal, a further reduction in EI values was observed, indicating higher red cell rigidity even though at the same time lactate concentration has a tendency to decrease.

The present study also demonstrated an alteration of the aggregation parameters illustrated by a significant increase in AI and a fall in *T*_1/2_ values. This is in agreement with some previous studies which have shown a similar tendency [4]. The mechanisms of these rheological changes are not clear but fibrinogen is the major plasma component responsible for red cell aggregation in blood [32]. In our study we observed increases in fibrinogen concentrations after exercise but the effect was transient and present only in one series. The changes in aggregation parameters were reversed in the postexercise period irrespective of the consumed meal and this was accompanied by significant decreases in fibrinogen concentrations in both groups.

In theory most alterations in blood rheology observed after acute exercise could exert negative effects particularly on the cardiovascular system and oxygen supply to the tissues. According to Poiseuille's law, a rise in blood viscosity could lead to increased vascular resistance and cardiac afterload [4]. However, some studies have shown that the effects of increased blood viscosity may be in healthy persons quite the opposite. Increased shear stress stimulates nitric oxide (NO) release from endothelium and leads to a decrease in vascular resistance [33, 34]. Connes et al. [32] have recently reported a positive correlation between a rise in blood viscosity and an increase in NO production during exercise and a negative correlation between an increase in blood viscosity and a decrease in vascular resistance. The increase in blood viscosity could be a physiological mechanism necessary for increased NO biosynthesis and adequate vasodilation. However, this physiological compensatory response could be effective only in normally functioning endothelium and in cases of endothelial dysfunction the increase in blood viscosity might be more detrimental to the cardiovascular system [35].

Nutritional factors could affect hemorheologic alterations associated with exercise. The most important factor is dehydration, which not only increases hematocrit, plasma osmolality, and blood and plasma viscosity but also red blood cell aggregation proportional to a rise in plasma globulin. Adequate hydration could almost completely prevent the increase in red cell rigidity induced by 1 hour of submaximal exercise [36].

There are a few reports about the effects of a preexercise meal on blood rheological responses to exercise. In a study by van der Brug et al. [37] no effects of different kinds of feedings on the hemorheological response to prolonged exercise have been found. Guezennec et al. [38] have shown that polyunsaturated fatty acids of the omega-3 family improved red cell flexibility. Brun et al. [11] tested the effects of breakfast eaten before a cycling session and found that a meal prevented a reduction in red cell deformability and increased plasma viscosity after exercise.

In conclusion, the present study demonstrated that high-intensity exercise causes several changes in blood rheology parameters, such as an increase in blood viscosity accompanied by hematocrit elevation, a decrease in EI and increase in AI, and a fall in *T*_1/2_ values. The meal eaten in the postexercise period caused a further reduction in EI values, indicating higher red cell rigidity, but not in plasma viscosity or aggregations indices. Such alterations in hemorheologic parameters should not impair cardiovascular system functions in healthy and fit people but could constitute a serious risk under various pathophysiological conditions.

## Figures and Tables

**Table 1:** Mean values (±SD) or median values (interquartile ranges) of plasma viscosity (PV), hematocrit (HCT), plasma lactate, and fibrinogen in meal series (*n* = 12).

Parameter	Basal	Exercise	Meal	*P*	*P*
				Basal versus exercise	Exercise versus meal
PV (mPa*·*s)	1.04 ± 0.1	1.3 ± 0.09	1.28 ± 0.18	0.000∗∗∗	0.808
HCT (%)	45.91 ± 1.1	49.26 ± 1.13	44.99 ± 1.31	0.000∗∗∗	0.000∗∗∗
Lactate (mmol/L)	1.32 ± 0.1	7.32 ± 0.81	5.43 ± 0.971	0.023∗	0.067∗
Fibrinogen (g/L)	3.41 (3.31–3.57)	3.79 (3.56–4.24)	3.14 (3.05–3.59)	0.004∗∗	0.003∗∗

*Significantly different (*P* < 0.05).

∗∗Significantly different (*P* < 0.001).

∗∗∗Significantly different (*P* < 0.001).

**Table 2:** Mean values (±SD) or median values (interquartile ranges) of plasma viscosity (PV), hematocrit (HCT), plasma lactate, and fibrinogen in control (without meal) series (*n* = 12).

Parameter	Basal	Exercise	Rest	*P*	*P*
				Basal versus exercise	Exercise versus rest
PV (mPa*·*s)	1.06 (1.01–1.08)	1.26 (1.22–1.32)	1.46 (1.33–1.54)	0.002∗∗	0.023∗
HCT (%)	47.55 ± 2.54	50.18 ± 0.96	48.24 ± 2.4	0.002∗∗	0.005∗∗
Lactate (mmol/L)	1.41 ± 0.1	7.45 ± 0.81	4.78 ± 1.2	0.037∗	0.069∗
Fibrinogen (g/L)	3.79 (3.54–4.81)	3.72 (3.62–3.92)	3.58 (3.35–3.88)	0.959	0.041∗

*Significantly different (*P* < 0.05).

∗∗Significantly different (*P* < 0.001).

**Table 3:** Mean values (±SD) or median values (interquartile ranges) of the total extent of aggregation (AMP), half time of total aggregation (*T*_1/2_), aggregation index (AI), and the elongation index (EI) at various levels of shear stress in meal series (*n* = 12).

Parameter	Basal	Exercise	Meal	*P*	*P*
				Basal versus exercise	Exercise versus meal
AMP (au)	18.31 (17.51–18.85)	18.65 (17.83–19.11)	17.62 (16.58–18.46)	0.347	0.05
*T*_1/2_ (s)	3.29 (2.98–3.48)	2.82 (2.7–2.97)	3.41 (3.24–3.91)	0.025∗	0.028
AI (%)	50.43 (49.53–52)	59.04 (58.21–59.78)	53.8 (49.14–56.08)	0.002∗∗	0.005∗∗
EI (0.3 Pa)	0.04 (0.03–0.05)	0.04 (0.03–0.07)	0.06 (0.06–0.07)	0.875	0.116
EI (0.58 Pa)	0.06 (0.05–0.08)	0.06 (0.05–0.1)	0.1 (0.06–0.11)	0.666	0.077
EI (1.13 Pa)	0.11 ± 0.03	0.1 ± 0.03	0.1 ± 0.03	0.034∗	1.000
EI (2.18 Pa)	0.23 (0.14–0.24)	0.21 (0.11–0.24)	0.2 (0.12–0.23)	0.003∗	0.505
EI (4.24 Pa)	0.34 (0.24–0.35)	0.32 (0.21–0.35)	0.3 (0.23–0.33)	0.015∗	0.125
EI (8.23 Pa)	0.45 (0.43–0.46)	0.42 (0.33–0.44)	0.41 (0.32–0.41)	0.002∗	0.014∗
EI (15.98 Pa)	0.52 (0.49–0.53)	0.51 (0.42–0.52)	0.46 (0.43–0.5)	0.025∗	0.026∗
EI (31.04 Pa)	0.58 (0.55–0.58)	0.57 (0.49–0.58)	0.53 (0.49–0.56)	0.135	0.004∗∗
EI (59.97 Pa)	0.61 (0.59–0.61)	0.6 (0.54–0.61)	0.59 (0.53–0.6)	0.13	0.008∗∗

*Significantly different (*P* < 0.05).

∗∗Significantly different (*P* < 0.001).

**Table 4:** Mean values (±SD) or median values (interquartile ranges) of the total extent of aggregation (AMP), half time of total aggregation (*T*_1/2_), aggregation index (AI), and the elongation index (EI) at various levels of shear stress in control (without meal) series (*n* = 12).

Parameter	Basal	Exercise	Rest	*P*	*P*
				Basal versus exercise	Exercise versus rest
AMP	18.74 ± 0.82	18.78 (18.32–19.52)	19.48 (17.98–19.99)	0.816	0.06
*T*_1/2_ (s)	3.86 ± 0.91	2.76 ± 0.41	3.68 ± 0.62	0.000∗∗∗	0.000∗∗∗
AI (%)	53.22 ± 5.82	59.45 ± 4.93	50.9 ± 5.15	0.001∗∗	0.000∗∗∗
EI (0.3 Pa)	0.05 (0.04-0.05)	0.06 (0.05-0.06)	0.05 (0.04-0.05)	0.068	0.059
EI (0.58 Pa)	0.07 ± 0.01	0.07 ± 0.01	0.08 ± 0.02	0.435	0.057
EI (1.13 Pa)	0.11 ± 0.02	0.1 ± 0.03	0.1 ± 0.02	0.058	0.966
EI (2.18 Pa)	0.22 (0.18–0.24)	0.2 (0.18–0.21)	0.19 (0.18–0.21)	0.019∗	0.41
EI (4.24 Pa)	0.33 (0.28–0.34)	0.31 (0.23–0.33)	0.3 (0.26–0.34)	0.045∗	0.695
EI (8.23 Pa)	0.44 (0.42–0.45)	0.43 (0.33–0.43)	0.41 (0.39–0.44)	0.002∗∗	0.53
EI (15.98 Pa)	0.52 (0.49–0.53)	0.5 (0.44–0.51)	0.48 (0.46–0.52)	0.023∗	0.695
EI (31.04 Pa)	0.57 (0.56–0.58)	0.56 (0.51–0.58)	0.55 (0.52–0.57)	0.016∗	0.929
EI (59.97 Pa)	0.6 (0.59–0.6)	0.59 (0.56–0.6)	0.59 (0.58–0.61)	0.084	0.594

*Significantly different (*P* < 0.05).

∗∗Significantly different (*P* < 0.001).

∗∗∗Significantly different (*P* < 0.001).

**Table 5:** Median values (interquartile ranges) of half-maximal shear stress (SS_1/2_), maximum elongation index (EI_max⁡_), and SS_1/2_/EI_max⁡_ ratio in meal series (*n* = 12).

Parameter	Basal	Exercise	Meal	*P*	*P*
				Basal versus exercise	Exercise versus meal
SS_1/2_ (Pa)	4.26 (3.873–5.267)	4.71 (4.174–6.706)	4.54 (4.146–6.444)	0.0342∗	0.9375
EI_max⁡_	0.66 (0.641–0.661)	0.65 (0.616–0.659)	0.62 (0.597–0.632)	0.2244	0.0907
SS_1/2_/EI_max⁡_	6.45 (5.918–8.532)	7.24 (6.320–11.592)	7.28 (6.713–11.195)	0.0022∗∗	0.8139

*Significantly different (*P* < 0.05).

∗∗Significantly different (*P* < 0.001).

**Table 6:** Median values (interquartile ranges) of half-maximal shear stress (SS_1/2_), maximum elongation index (EI_max⁡_), and SS_1/2_/EI_max⁡_ ratio in control (without meal) series (*n* = 12).

Parameter	Basal	Exercise	Rest	*P*	*P*
				Basal versus exercise	Exercise versus meal
SS_1/2_ (Pa)	4.18 (4.028–5.193)	4.94 (4.765–5.872)	4.68 (4.418–5.265)	0.0022∗∗	0.1579
EI_max⁡_	0.65 (0.645–0.658)	0.65 (0.622–0.659)	0.64 (0.611–0.659)	0.4240	0.6234
SS_1/2_/EI_max⁡_	6.38 (6.150–8.026)	7.54 (7.133–9.878)	7.53 (6.731–8.550)	0.0022∗∗	0.1823

**Significantly different (*P* < 0.001).
